# *Pseudomonas* bacteriocin syringacin M released upon desiccation suppresses the growth of sensitive bacteria in plant necrotic lesions

**DOI:** 10.1111/1751-7915.13367

**Published:** 2019-01-22

**Authors:** Jun‐Zhou Li, Li‐Ying Zhou, You‐Liang Peng, Jun Fan

**Affiliations:** ^1^ Ministry of Agriculture Key Laboratory for Crop Pest Monitoring and Green Control China Agricultural University Beijing 100193 China; ^2^ State Key Laboratory of Agrobiotechnology China Agricultural University Beijing 100193 China; ^3^ Joint Laboratory for International Cooperation in Crop Molecular Breeding Ministry of Education China Agricultural University Beijing 100193 China

## Abstract

Bacteriocins are regarded as important factors mediating microbial interactions, but their exact role in community ecology largely remains to be elucidated. Here, we report the characterization of a mutant strain, derived from *Pseudomonas syringae* pv. *tomato* DC3000 (*Pst*), that was incapable of growing in plant extracts and causing disease. Results showed that deficiency in a previously unannotated gene *saxE* led to the sensitivity of the mutant to Ca^2+^ in leaf extracts. Transposon insertions in the bacteriocin gene *syrM*, adjacent to *saxE*, fully rescued the bacterial virulence and growth of the *ΔsaxE* mutant in plant extracts, indicating that *syrM‐saxE* encode a pair of bacteriocin immunity proteins in *Pst*. To investigate whether the *syrM‐saxE* system conferred any advantage to *Pst* in competition with other SyrM‐sensitive pathovars, we compared the growth of a SyrM‐sensitive strain co‐inoculated with *Pst* strains with or without the *syrM* gene and observed a significant *syrM‐*dependent growth reduction of the sensitive bacteria on plate and in lesion tissues upon desiccation–rehydration treatment. These findings reveal an important biological role of SyrM‐like bacteriocins and help to understand the complex strategies used by *P. syringae* in adaptation to the phyllosphere niche in the context of plant disease.

## Graphical abstract

SyrM confers advantage to the producer in competition with other sensitive bacteria upon desiccation. Our findinds reveal an important biological role that bacteriocin played during adaptation to the phyllosphere niche in the context of plant disease.

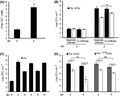

## Introduction

Plant leaves make up the majority of phyllosphere and are inhabited by highly complex bacterial communities consisting of both commensal and adversary species (Vorholt, 2012). The relative abundance of phyllosphere bacterial populations is often conditioned collectively by multiple factors including host plants, environmental cues and interactions between diverse microbial species (Hirano and Upper, 2000; Vorholt, 2012; Wagner *et al*., 2016). When an appropriate relative abundance is achieved, opportunistic pathogens, such as *Pseudomonas syringae* species, may launch a range of molecular weapons including phytotoxins and proteinaceous effectors to subvert host immunity and manipulate plant physiology in favour of disease (Nomura *et al*., 2006; Zheng *et al*., 2012; Xin *et al*., 2016) or frost injury (Lindow *et al*., 1982). Current understanding on the molecular basis of how *P. syringae* bacteria predominate the phyllosphere niche prior to the attacks is still limited. Early observations reveal that host‐adapted *P. syringae* survives better than non‐pathogenic bacteria on leaf surface under UV irradiation or desiccation (Wilson *et al*., 1999), whereas Lee *et al*. (2012) found that type three effectors of *P. syringae* pv. *syringae* B728a influence the survival and size of the foliar epiphytic bacterial populations (Lee *et al*., 2012). Microbial competitions involving rapid exploitation of the limiting resource(s) or antibiosis are also likely employed in the complex interactions among bacterial populations coexisting in the phyllosphere. The exploitation competition has been widely exhibited in studies of biological control of detrimental *P*. *syringae* strains (Lindow, 1987; Völksch and May, 2001; Wensing *et al*., 2010; Xie *et al*., 2017); in contrast, the significance of antibiosis is not well established for bacterial competition with *P. syringae* on leaf surfaces (Lindow and Brandl, 2003). For instance, the production of three antimicrobial compounds in the biocontrol strain Pss22d is dispensable for its antagonistic effect *in planta* against *P*. *syringae* pv. *glycinea* that causes bacterial blight of soybean (Braun *et al*., 2010).

Bacteriocins are bacteria‐secreted proteins that are able to kill the close relatives of the producing strain and are regarded as potential competition factors in microbial ecosystems (Riley and Wertz, 2002). Colicin M‐like bacteriocins are a class of bacteriocins that share high homology with the Colicin M (ColM), a bacteriocin originally identified from *Escherichia coli* strains and can kill the sensitive bacteria by interfering cell wall synthesis. The mode of action of ColM has been well characterized in *E. coli*. Extracellular ColM needs to be taken up by an outer membrane receptor and traverses the outer membrane via the TonB‐dependent pathway to reach the periplasm, in which it degrades the substrate lipid II, an important precursor used for the biosynthesis of peptidoglycan, thereby inhibits the cell wall synthesis and triggers bacterial cell death (Barreteau *et al*., 2012). The expression of ColM is tightly regulated by SOS promoters. Thus, under normal conditions, the ColM encoding gene *cma* is tightly repressed by the LexA protein, and under SOS conditions, the RecA protease is activated and cleaves the LexA repressor protein, which activates the expression of a group of SOS genes including *cma* (Cascales *et al*., 2007). ColM producers can protect themselves by expression of the cognate immunity protein Cmi (Ölschläger *et al*., 1991; Gérard *et al*., 2011). ColM‐like bacteriocins have been identified from a range of bacterial species including *P*. *syringae* (Barreteau *et al*., 2009; Grinter *et al*., 2012; Ghequire and De Mot, 2015), and their biological role in bacterial competition has not been elucidated, although many of them have been shown to exhibit lipid II‐cleaving activity and inhibit the growth of close relatives in culture (Barreteau *et al*., 2009; Grinter *et al*., 2012; Ghequire and De Mot, 2015).

During our previous work searching for bacterial *sax* (*s*urvival in *A* *rabidopsis* e*x*tracts) genes that protect *P*. *syringae* against plant‐released antimicrobials, transposon Ω‐Km was used to mutagenize the bacteria, and a number of mutant strains sensitive to plant extracts were isolated. Molecular characterization of the mutants reveals that multiple *sax* genes (*saxCAB/F/D/G*) encoding the metabolic enzyme and multidrug efflux pumps are required for *P. syringae* to overwhelm isothiocyanate‐based defence during the necrotrophic stage of infection of *Arabidopsis* plants (Fan *et al*., 2011). Here, we report the molecular characterization of another resulting *sax* mutant that failed to grow on plant extracts and was unable to cause disease *in planta*. The investigation revealed that a previously unannotated gene, *saxE*, acted as an immunity gene of the ColM‐like bacteriocin syringacin M (SyrM) in *P. syringae* pv. *tomato* DC3000 (*Pst*) strain and prevented bacteria from being killed by SyrM in the presence of calcium ion. Furthermore, we demonstrated that the *syrM‐saxE* system was required for *Pst* to suppress the population of SyrM‐sensitive *P. syringae* during desiccation–rehydration cycles. Our findings reveal an important biological role of SyrM‐like bacteriocins and highlight the capacity of *P*. *syringae* to integrate environmental stresses into the complex bacterial interactions for maintaining a prevailing population in the context of plant disease.

## Results

### Isolation of a *Pst* mutant strain that was unable to grow in leaf extracts and to cause disease

**Figure 1:**
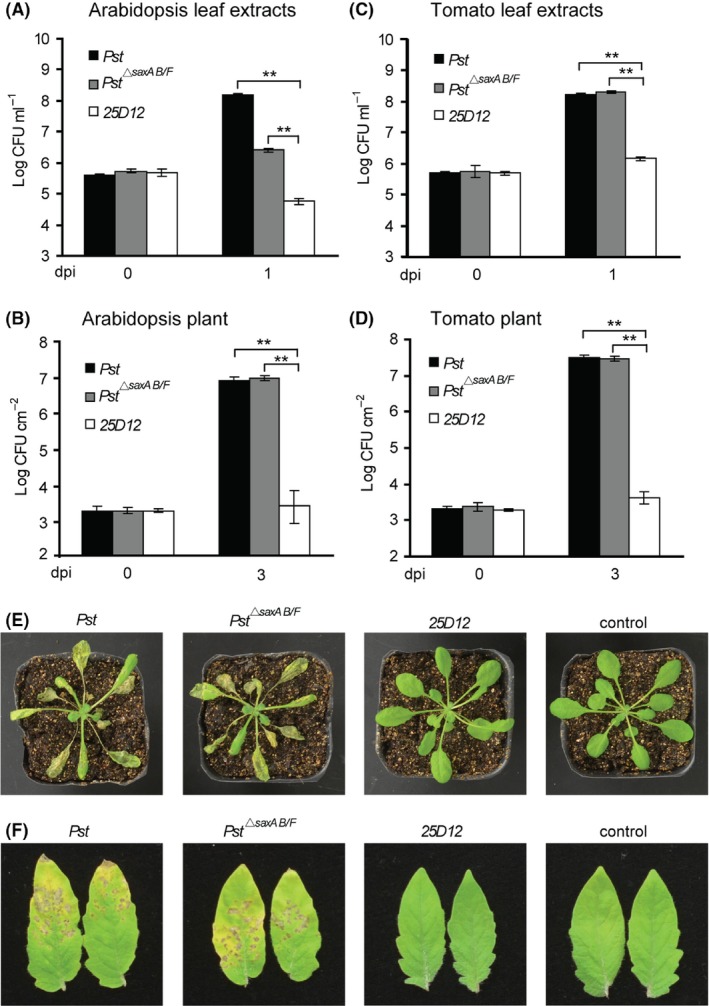
*Pst* mutant *25D12* was sensitive to plant extracts and unable to cause disease. For the growth in leaf extracts assay, wild‐type *Pst*,* Pst* ^*▵sax*^ ^*AB*^ ^*/F*^ and *25D12* strains were inoculated into the 100% *Arabidopsis* (A) and tomato (C) leaf extracts at OD_600_ = 0.001. Samples were collected at 0 and 1 day postinoculation (dpi) for colony counts. For the growth *in planta* assay, bacterial strains were infiltrated into *Arabidopsis* (B) and tomato (D) leaves at OD_600_ = 0.001. Samples were taken at 0 and 3 dpi for colony counts. *Arabidopsis* (E) and tomato (F) plants were sprayed with the bacteria at OD_600_ = 0.2, and disease symptoms were photographed 4 days after inoculation. Non‐sprayed plants were used as control. All experiments were repeated at least three times, and similar results were observed. Data shown are means ± SD. ** indicates statistical significance (*t*‐test, *P *<* *0.01).

Our previous studies have revealed that the *Pst* ^*▵saxAB/F*^ strain, in which an isothiocyanate‐metabolizing enzyme and a multidrug efflux system were disrupted, was partially compromised for bacterial growth in *Arabidopsis* leaf extracts. To seek additional *sax* genes, transposon‐mutagenized clones derived from the *Pst* ^*▵saxAB/F*^ strain were screened for enhanced sensitivity to the plant extracts (Fan *et al*., 2011). Out of ~4000 clones, we obtained a mutant strain (*25D12*) that failed to grow in *Arabidopsis* extracts (Fig. 1A) but grew well in rich media (Fig. S1). Subsequent disease assay showed that there was no increase in the population of *25D12* when infiltrated into *Arabidopsis* leaves (Fig. 1B), and no disease symptom could be observed on spray‐inoculated plants compared to the wild‐type *Pst* or the parental *Pst* ^*▵saxAB/F*^ strain (Fig. 1E). When tomato plants, the natural host of *Pst*, were inoculated with the *25D12* mutant, similar levels of defects in bacterial growth and virulence could be observed (Fig. 1C, D and F). These findings indicate that: (i) in *25D12* mutant strain, a genetic component was compromised, which functions either alone or with other *sax* genes to support the survival and virulence of *Pst* during infection; and (ii) the factor(s) in leaf extracts that inhibited the growth of *25D12* mutant may exist in species of diverse plant families.

### Identification of a previously unannotated gene necessary for *Pst* to grow in extracts and *in planta*

**Figure 2:**
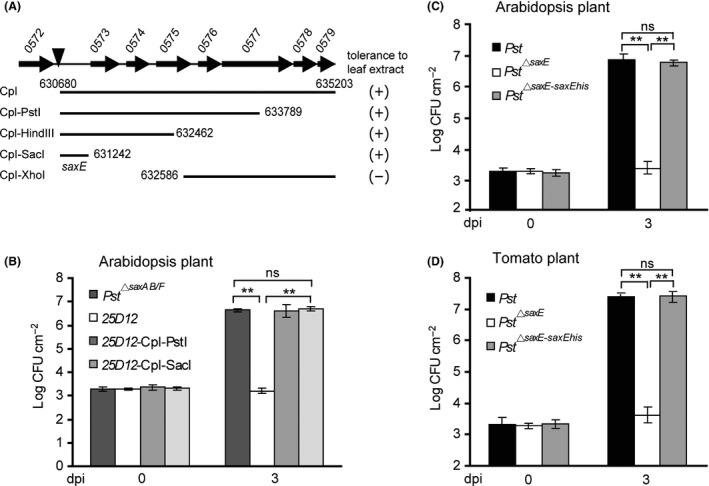
The *saxE* gene was required for *Pst* growth in leaf extracts and *in planta*. A. A schematic diagram of the complementary tests pinpointing the minimal region required for rescuing the growth of *25D12* mutant in plant leaf extracts. Filled arrows indicate genes annotated by the sequencing project marked with locus tags on the top. The filled triangle shows the insertion site of Ω‐Km transposon in mutant *25D12*. Genomic DNA fragments downstream the transposon insertion site were tested for the ability to confer tolerance to leaf extracts. B. The minimal region that protected *25D12* in leaf extracts also rescued the growth of the mutant on *Arabidopsis* plant. Bacterial inocula were infiltrated into *Arabidopsis* leaves at OD_600_ = 0.001. Samples were taken at 0 and 3 dpi for colony counts. The *saxE orf* was required for bacterial virulence on *Arabidopsis* (C) and tomato (D) plants. Wild‐type *Pst*,* Pst* ^*▵saxE*^ and the complemented strain *Pst* ^*▵saxE*‐*saxEhis*^ were infiltrated into *Arabidopsis* (C) and tomato (D) leaves at OD_600_ = 0.001. Samples were taken at 0 and 3 dpi for colony counts. All experiments were repeated at least three times, and similar results were observed. Data shown are means ± SD. ** indicates significant difference (*t*‐test, *P *<* *0.01). ns indicates no significant difference (*t*‐test, *P *>* *0.05).

Results of TAIL‐PCR showed that the transposon in *25D12* mutant was inserted in a cluster of annotated genes between *PSPTO_0572* and *PSPTO_0573* (Fig. 2A). DNA fragments downstream the transposon insertion site were thus used to complement the *25D12* mutant, and a minimal region of 562 base pairs of DNA (Cpl‐SacI) containing no annotated gene was unexpectedly found capable of restoring bacterial growth in leaf extracts and *in planta* (Fig. 2A and B). Further sequence analysis detected several open reading frames (*orfs*) in the 562 base‐pair region, and the longest one (designated as *saxE* hereafter) encodes a deduced protein of 135 amino acids (Fig. 4A) similar to a group of hypothetical proteins from several species of *Pseudomonas* bacteria (Table S1).

**Figure 3:**
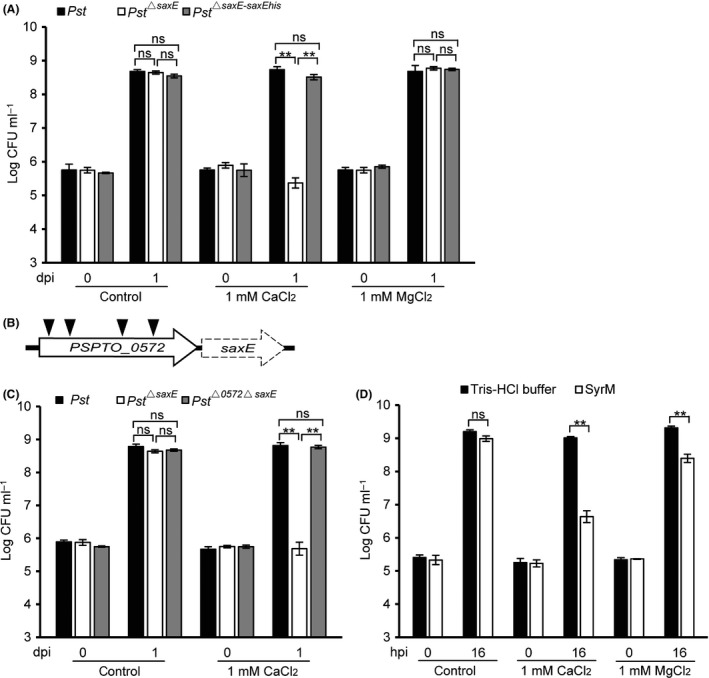
Inhibitory effect of Ca^2+^ on the growth of strains deficient in the *saxE* gene. A. Ca^2+^ is the inhibitory factor that suppressed the growth of *Pst* ^*▵saxE*^. Bacteria were inoculated into the King's B (KB) liquid medium supplemented with 1 mM CaCl_2_ or MgCl_2_ at OD_600_ = 0.001. Samples were collected at 0 and 1 dpi for colony counts. B. A schematic diagram of Ω‐Km transposon insertion events that suppressed the *Pst* ^*▵saxE*^ phenotype. Filled triangles indicate the insertion sites that were able to restore the growth of *Pst* ^*▵saxE*^ in the presence of Ca^2+^. The empty and the dashed arrows denote the gene *PSPTO_0572 (syrM)* and the deleted *saxE* gene respectively. C. Deletion of *syrM* abolished the growth inhibitory effect of Ca^2+^ on *Pst* ^*▵saxE*^. Bacteria were inoculated into the KB liquid medium supplemented with 1 mM CaCl_2_ at OD_600_ = 0.001, and samples were collected at 0 and 1 dpi for colony counts. D. Ca^2+^ and Mg^2+^ enhanced inhibitory activity of bacteriocin SyrM on the sensitive *P. syringae* pv. *lachrymans*‐8 (*Psl*). Purified SyrM (1 μM) was mixed with 100 μl KB liquid cultures supplemented with 1 mM CaCl_2_ or MgCl_2_ inoculated by *Psl* at OD_600_ = 0.0002. Samples were collected at 0 and 16 h postinoculation (hpi) for colony counts. Plain KB medium was used as control. All experiments were repeated at least three times, and similar results were observed. Data shown are means ± SD. ** indicates significant difference (*t*‐test, *P *<* *0.01). ns indicates no significant difference (*t*‐test, *P *>* *0.05).

**Figure 4:**
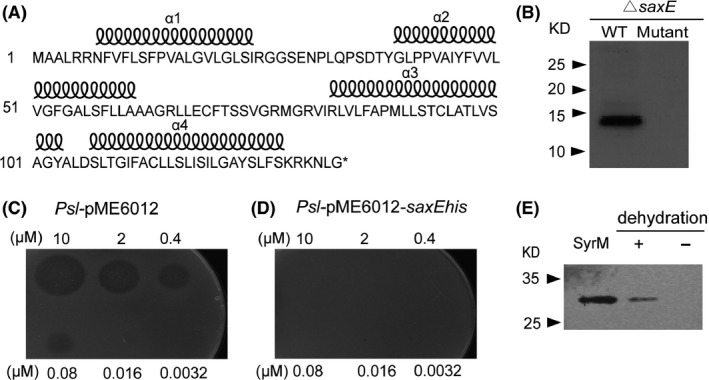
The *saxE* gene encodes an immunity protein of the bacteriocin SyrM. A. Amino acid sequence and the predicted secondary structure of SaxE protein. The coils above the amino acids denote the helices predicted by TMHMM (http://www.cbs.dtu.dk/services/ TMHMM/). B. Gel blot assay of the SaxE protein. Proteins extracted from *Pst* ^*▵saxE*^ bacteria harbouring the pME6012 expressing wild‐type SaxEhis (WT) or mutated SaxEhis(ins) (mutant) protein were used for blot assay detected with the his‐tag antibody. C, D. Purified SyrM protein inhibited the growth of *Psl*. Five microlitres of serial‐diluted SyrM protein solutions was applied on a plate overlaid with soft agar inoculated with *Psl*‐pME6012 (C) or *Psl*‐pME6012‐*saxEhis* (D). The plates were incubated at 28°C and photographed 1 day after inoculation. E. SyrM is released upon desiccation treatment. *Pst*‐pME6012‐*syrMhis* (OD_600_ = 0.01) was applied onto filter paper discs placed on KB plates and cultured at 28°C for 16 h. The discs were desiccated for 4 h (+), and non‐treated samples were used as control (−). Bacteria were washed off from the discs and spun, and the resulting supernatant samples, together with the purified SyrM protein, were used for gel blot assay with the his‐tag antibody.

To investigate whether the *saxE orf* truly encodes a functional protein, we deleted the entire coding region of *saxE* from the wild‐type *Pst* by homologous recombination. The resulting *Pst* ^*▵saxE*^ mutant grew well in rich media (Fig. 3A) but failed to grow in plant extracts and during infection (Fig. 2C, D and S2A), indicating that the loss of *saxE* alone was responsible for the growth defects in *25D12* strain. We modified the Cpl‐SacI DNA fragment by fusing a his‐tag to the C‐terminus of the predicted SaxE protein (*saxEhis*) and also introduced one nucleotide into the coding region of *saxEhis* to generate the *saxEhis(ins)* construct, leading to a frameshift only in the *saxE* but not other smaller *orfs* on the DNA fragment. Plasmids harbouring these modified inserts were delivered into the *Pst* ^*▵saxE*^ mutant, and resultant strains were subjected to growth assay. Results showed that constructs bearing *saxEhis* but not *saxEhis(ins)* were able to restore the growth of the *Pst* ^*▵saxE*^ mutant in 100% plant extracts and i*n planta* (Fig. 2C, D and S2A), suggesting that the *saxE* gene played a key role in protecting bacteria in plant extracts and *in planta*. The protein gel blot assay detected a protein of the same size as SaxEhis in the strain *Pst* ^*▵saxE*^ complemented with *saxEhis* (Fig. 4B), which corroborated that *saxE* encodes a protein essential for the growth of *Pst* in plant extracts and *in planta*.

### Ca^2+^ was the inhibitory component in plant extracts that suppressed the growth of *Pst* ^*▵saxE*^ strain

*Pseudomonas sax* genes have been shown crucial for *Pst* to overcome the aliphatic isothiocyanate‐based defence in *Arabidopsis* (Fan *et al*., 2011). However, tomato plants have not been reported as a source of these metabolites, and we speculated that additional factor in plant extracts conferred the inhibitory effect on *Pst* ^*▵saxE*^ strain. Thus, by using ion exchange chromatography we isolated the inhibitory components from leaf extracts and crystallized the active eluates with methanol (see details in Experimental procedures). Nevertheless, the nuclear magnetic resonance (NMR) analysis of these crystals only detected a methyl proton peak (at 1.8 ppm) and two carbon peaks (a strong methyl peak at 23 ppm and a weaker quaternary peak at 181 ppm), reminiscent of the spectra of the acetate ion. Since 10% acetic acid was used for elution during chromatography, we speculated that a metal cation might be the active agent in the crystal. We further tested a panel of metal acetates and found that only calcium acetate preferentially inhibited the bacterial growth of *Pst* ^*▵saxE*^ strain (Fig. S2B). We thus dissolved the crystal in water and measured the Ca^2+^ levels of the solution, and the calcium content of the crystal was thereby determined to be 20.1% (w/w), close to that of Ca(C_2_H_3_O_2_)_2_·H_2_O, which is 22.7% (w/w), indicating that the crystal mainly consists of calcium acetate. Subsequent analysis confirmed that 1 mM of Ca^2+^ but not Mg^2+^, another divalent cation abundant in plants, strongly inhibited the growth of *Pst* ^*▵saxE*^ in rich media, and this inhibition was abolished on the mutant complemented with the wild‐type *saxE* gene (Fig. 3A). These observations demonstrated that wild‐type *Pst* strain requires *saxE* for protection against the Ca^2+^.

### The inhibitory effect of Ca^2+^ on *Pst* ^*▵saxE*^ strain was dependent on the bacterial gene *PSPTO_0572*

To further dissect the mechanism underlying the inhibitory effect of Ca^2+^ on the *Pst* ^*▵saxE*^ strain, transposon‐mutagenized *Pst* ^*▵saxE*^ bacteria were thus subjected to screen for revertant colonies grown on plates supplemented with 10 mM of CaCl_2_. Thirteen clones were isolated from the screen and TAIL‐PCR revealed that only the locus *PSPTO_0572* was disrupted independently in four clones (Fig. 3B), indicating that this locus is required for the Ca^2+^‐mediated growth suppression of *Pst* ^*▵saxE*^ strain. Indeed, the bacterial growth in the presence of 1 mM of CaCl_2_ was completely restored when *PSPTO_0572* was deleted from the *Pst* ^*▵saxE*^ background (Fig. 3C). Moreover, by quantitative RT‐PCR analysis, we observed a modest increase in transcript levels of *PSPTO_0572* in wild‐type *Pst* strain upon Ca^2+^ treatment, whereas the increase was more prominent in the *Pst* ^*▵saxE*^ strain following the same treatment (Fig. S3).

The locus *PSPTO_0572* encodes a ColM‐like bacteriocin, SyrM (Grinter *et al*., 2012). Our observations indicated that Ca^2+^ may be a potent enhancer of this bacteriocin and *saxE* may function as its immunity gene. We thus expressed the his‐tagged SyrM in *E. coli* and used the purified protein to test the bacteriocin activity on 16 *P*.* syringae* strains. Results showed that the growth of pathovars *lachrymans* (8 and NCPPB540) and *glycinea* (race 4) was suppressed by SyrM treatment (Table S2, Fig. 4C), and the inhibitory effect increased significantly by the addition of 1 mM of CaCl_2_ or MgCl_2_ (Fig. 3D). We further transformed *P. syringae* pv. *lachrymans*‐8 (*Psl*) with the plasmid carrying the *saxE* gene from *Pst*, and the resulting *Psl‐saxE* strain was able to resist the highest tested concentration (10 μM) of SyrM (Fig. 4D), demonstrating that the *Psl‐saxE* strain has gained full immunity to the bacteriocin.

### The SyrM‐mediated bacterial competition upon dehydration–rehydration treatment

**Figure 5:**
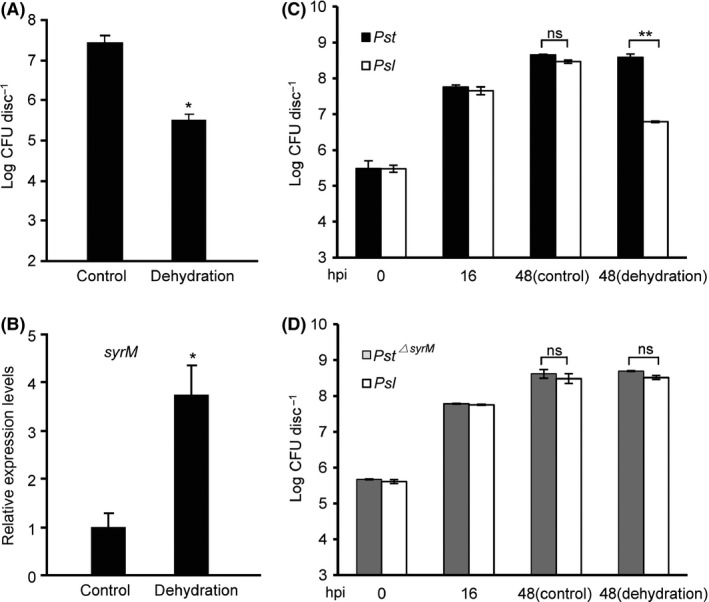
SyrM‐mediated competition between *Pst* and *Psl* on plate culture. A. Desiccation treatment reduced the number of viable bacteria on filter paper discs. Wild‐type *Pst* (OD_600_ = 0.01) was applied onto filter paper discs placed on plates and cultured for 16 h. The inoculated filter paper discs were desiccated for 4 h. Desiccated and untreated paper discs were collected for bacterial counts. B. Total RNA samples were prepared from bacteria on paper discs collected in (A) and subjected to the quantitative real‐time PCR assay of relative transcript levels of *syrM*. The *recA* was used as the reference gene. C, D. SyrM‐mediated competition depended on dehydration and rehydration treatments. The SyrM‐sensitive *Psl* were mixed at 1:1 ratio with wild‐type *Pst* (C) and *Pst* ^*▵syrM*^ (D) strains, respectively, and co‐cultured at the same conditions as in (A). After desiccation treatment, the paper discs were rehydrated and cultured to 48 h. Untreated samples were used as controls. All experiments were repeated at least three times, and similar results were observed. Data shown are means ± SD. ** indicates significant difference (*t*‐test, *P *<* *0.01). * indicates significant difference (*t*‐test, *P *<* *0.05). ns indicates no significant difference.

The role of ColM or ColM‐like bacteriocins in bacterial competition has not been described experimentally, although the bactericidal activity of purified proteins on sensitive strains has been demonstrated (Braun *et al*., 1974; Barreteau *et al*., 2009). To investigate the potential impact of *syrM* locus on sensitive bacteria, we co‐cultured *Psl* with wild‐type *Pst* and the mutant *Pst* ^*▵syrM*^, respectively, on filter paper discs. Since the expression of *syrM* appears to be regulated by an SOS promoter, we initially cultured bacteria under SOS‐inducing conditions including UV‐B or mitomycin C (MMC) treatments, but did not find any difference in *Psl* population sizes between co‐cultures with *Pst* and *Pst* ^*▵syrM*^ strains (not shown). However, we found that a 4‐h dehydration treatment of the overnight culture was able to reduce *Pst* levels by nearly two orders of magnitude (Fig. 5A) and upregulate the expression of *syrM* accordingly (Fig. 5B). More importantly, when the desiccated paper discs were further cultured on a fresh plate for another day, population sizes of *Pst* and *Pst* ^*▵syrM*^ rebounded to similar levels, whereas the growth of *Psl* was significantly reduced when co‐cultured with *Pst* but not with *Pst* ^*▵syrM*^ (compare Fig. 5C and D), indicating the existence of SyrM‐mediated suppression of the sensitive bacteria. Since no difference could be observed in bacterial growth without desiccation treatment (Fig. 5C and D), we further examined whether the desiccation treatment facilitated the release of the bacteriocin from a *Pst* strain expressing the his‐tagged SyrM. Protein gel blot assays of the supernatants of bacterial wash‐offs detected a band of SyrM from desiccated paper discs but not from the untreated control (Fig. 4E), clearly suggesting a desiccation‐associated release of SyrM into the extracellular space.

### SyrM played a key role in suppression of the sensitive bacterial strain in lesion tissue

**Figure 6:**
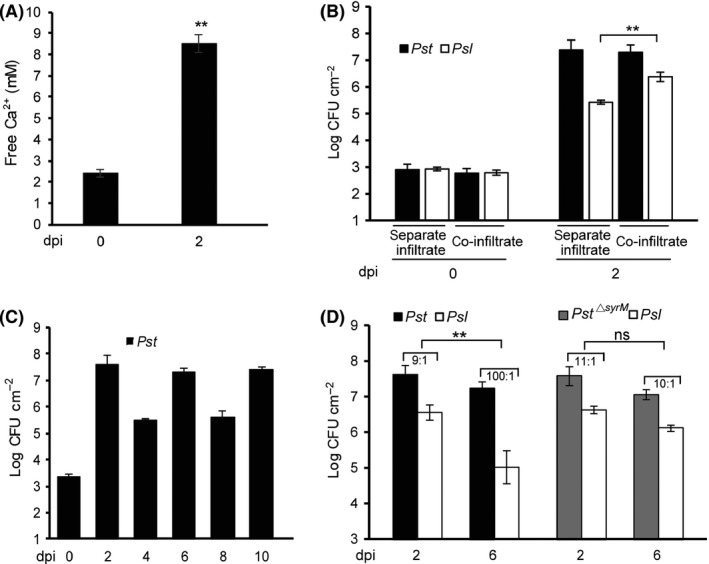
SyrM suppressed the rebound of *Psl* population during the rehydration of desiccated leaf lesions. A. Ca^2+^ released at the late stage of plant–bacteria interaction. *Pst* was infiltrated into tomato leaves at OD_600_ = 0.001. Leaves were collected at 0 and 2 dpi for detection of the concentration of free Ca^2+^ in the intercellular fluids. B. *Pst* infection promoted the growth of *Psl* on tomato plants. *Pst*,* Psl* and a 1:1 mixture of *Pst* and *Psl* were infiltrated into tomato leaves at OD_600_ = 0.0002 respectively. C. Bacterial population in leaf lesions fluctuated widely during a dehydration–rehydration regimen. *Pst* was infiltrated into tomato leaves at OD_600_ = 0.001. At 2 dpi, the inoculated leaf lesions were collected and desiccated for 2 day. After dehydration treatment, the desiccated leaf lesions were rehydrated for 2 day. The rehydrated leaf lesions were then treated again as the same desiccation–rehydration treatment. D. *syrM* is required for *Pst* to limit the growth of *Psl* in rehydrated lesion tissues. The 1:1 mixtures of *Pst/Psl* and *Pst* ^*▵syrM*^/*Psl* were infiltrated into tomato leaves respectively. At 2 dpi, the leaf lesions were collected and treated the same as in (C). Lesion samples were taken at 2 and 6 dpi for bacterial counts. The ratio above the columns is means of the ratio between two pathovars in lesion samples. All experiments were repeated at least three times, and similar results were observed. Data shown are means ± SD. ** indicates significant difference (*t*‐test, *P *<* *0.01). ns indicates no significant difference.

To further explore whether the observed *syrM*‐mediated competition may also occur *in planta*, we first measured the Ca^2+^ levels of the intercellular fluid during the *Pst* infection of tomato plants. Results showed that Ca^2+^ levels reached > 8 mM at 2 dpi when the water‐soaking lesions appeared, about fourfold of the basal levels at 0 dpi, indicating that Ca^2+^ was released into the extracellular space where bacteria colonize (Fig. 6A). We then inoculated tomato healthy plants with a 1:1 mixture of *Pst* and *Psl*, and compared the sizes of bacterial populations with separate inoculation of individual strains. Results showed that at 2 dpi *Psl* population in the leaves was about 10‐fold higher when co‐inoculated with *Pst* in comparison with inoculated by *Psl* alone (Fig. 6B). Hence, no competition between the two populations could be observed during the biotrophic stage of infection. However, when the lesion tissues collected at 2 dpi were repeatedly subjected to first dry conditions (32.5% RH) for 2 days and then wet conditions (100% RH) for 2 days, a fluctuation of nearly 2 orders of magnitude in *Pst* population could be observed (Fig. 6C). We therefore measured the ratio of *Pst*/*Psl* under this condition in comparison with that of *Pst* ^*▵syrM*^/*Psl*. As shown in Fig. 6D, at 2 dpi, the ratio of *Pst*/*Psl* (9:1) was similar to that of *Pst* ^*▵syrM*^/*Psl* (11:1), whereas at 6 dpi, after one cycle of dry–wet treatment, the ratio of *Pst*/*Psl* increased to about 100:1 and that of *Pst* ^*▵syrM*^/*Psl* still stayed around 10:1, similar to that of 2 dpi. These observations demonstrated that *Pst* was capable of suppressing the SyrM‐sensitive bacteria during the necrotrophic stage of infection.

## Discussion

Bacteriocins have been widely inferred as important factors mediating interactions between bacterial populations; however, the exact biological role of most bacteriocins remains to be elucidated (Riley and Wertz, 2002). A few studies have demonstrated that colicins participate in the bacterial competition. For instance, ColIb, a pore‐forming colicin, has been shown to confer a competitive advantage in inflammation‐induced overgrowth on a human pathogenic strain of *Salmonella enterica* against the commensal *E. coli* (Nedialkova *et al*., 2014). Nevertheless, the role of ColM or ColM‐like bacteriocins in bacterial community interactions has yet to be unveiled. In this study, we have identified the immunity gene of a ColM‐like bacteriocin SyrM in *P. syringae* and demonstrated that this bacteriocin is required for the suppression of sensitive bacteria in leaf lesions under a wet–dry–wet regimen (Fig. 6D). Phyllosphere bacteria have to endure the frequent environmental stress caused by desiccation, and it has been well documented in early studies that the size of the epiphytic bacterial population is dependent on the ambient moisture (And and Upper, 1983; Beattie, 2011). Indeed, we observed in the detached leaf lesions a sharp reduction of *P. syringae* populations under low ambient humidity, and that after rehydration, bacterial numbers rebound to levels similar to those before desiccation treatment (Fig. 6C and S4). The ability to restore the population during desiccation–rehydration cycles is essential for bacteria to colonize the epiphytic habitat and to poise a secondary infection (Leben, 1981; Lindow *et al*., 1993; Beattie and Lindow, 1994; Wilson *et al*., 1999; Quesada *et al*., 2010). Bacterial aggregate formation on the leaf surface is known to help to cope with desiccation (Beattie and Lindow, 1999; Vorholt, 2012). Previous reports demonstrated that the battle of those cells that were in direct contact with other strains was much more likely intense than that with the same strain in the aggregate (Monier and Lindow, 2005,b), in which antibiosis might mediate the bacterial interaction. It is still not clear how *P. syringae* strains managed the nutrient supply required for the regrowth in lesion tissues (Fig. 6C and S4), but suppression of closely related bacteria with bacteriocin such as SyrM (Fig. 6D) may help to compete for the limited nutrients. ColM‐like bacteriocin has been found in a wide range of bacterial species including *P. syringae* (Ghequire *et al*., 2018). Interestingly, the sequences of SyrM‐like proteins from *P. syringae* species are highly conserved (> 94% of identity), whereas they share much lower similarity with ColM‐like proteins from other *Pseudomonas* or non‐*Pseudomonas* species (Table S3), indicating that the SyrM‐mediated competition may be important for *P. syringae* strains in defining their particular ecological niche. In this study, we have investigated the impact of *Pst*‐produced SyrM on the population of a single sensitive strain and, more importantly, identified the key role of desiccation–rehydration cycles in exerting the impact of SyrM bacteriocin. As the interactions among phyllosphere bacterial communities are highly complex in nature (Vorholt, 2012), it is therefore intriguing to investigate further the potential influence of this class of bacteriocins on the structure of phyllosphere microbial communities.

The expression of most colicins is regulated by the SOS promoter (Cascales *et al*., 2007), and we have observed that MMC strongly enhanced the transcript levels of *syrM* in *Pst* (data not shown). However, when co‐cultured with *Pst*, the growth of *Psl* was not suppressed in either liquid or solid medium containing MMC at levels activating the expression of *syrM* (data not shown). This lack of suppression could be due to the failure of the release of SyrM protein. It has been shown that the release of some colicins is regulated by colicin release protein (CRP)‐mediated cell lysis (Wal *et al*., 1995; Cascales *et al*., 2007) or during the course of phage lysis (Nedialkova *et al*., 2016). As no SyrM‐associated lysis gene was identified in *Pst*, we thus speculate that this bacteriocin is released through an alternative mechanism which is still obscure at present. In this study, we observed a mild upregulation of *syrM* expression and a subsequent release of the protein into the extracellular space upon desiccation treatment (Figs 4E and 5B), and that at the same time, the levels of culturable *Pst* dropped about two orders of magnitude (Fig. 5A), implying that the release of SyrM was most likely associated with bacterial cell death. Apart from its impact on the gene activation and the release of bacteriocin protein, the dehydration stress might further concentrate the SyrM and enhance the inhibitory effect of the bacteriocin (Chao and Levin, 1981); whether the uptake of SyrM by the sensitive *Psl* was also enhanced during the dehydration and rehydration cycle remains to be elucidated.

It was unexpected to find that Ca^2+^ was the active agent inhibiting the growth of *Pst* ^*▵saxE*^ strain. Indeed, we observed a mobilization of Ca^2+^ into the extracellular fluid (8 mM) at a late stage of bacterial infection (Fig. 6A), and the actual Ca^2+^ levels in the lesion tissue could be much higher during desiccation. In addition, we also found that Ca^2+^, which has not been regarded as an SOS inducer, activated and enhanced the expression of *syrM* gene, especially in *Pst* ^*▵saxE*^ strain (Fig. S3). Previous reports have demonstrated that divalent cations including Ca^2+^ and Mg^2+^ are capable of enhancing the enzymatic activity of ColM or SyrM on degrading lipid II (Schaller *et al*., 1981a,b; Barreteau *et al*., 2009; Grinter *et al*., 2012). Consistent with these findings, our data showed that these cations could enhance the *in vitro* bactericidal activity of SyrM on *Psl* (Fig. 3D). However, the growth of *Pst* ^*▵saxE*^ strain was inhibited only by Ca^2+^ but not by Mg^2+^ (Fig. 3A). Similarly, Ca^2+^ but not Mg^2+^ has been found to boost the ColM‐induced cell lysis in liquid culture (Schaller *et al*., 1981a,b). These observations collectively suggested that factors other than the enzyme activity may account for the Ca^2+^‐dependent inhibitory effect. A possible explanation is that Ca^2+^ may enhance the uptake of the ColM‐like bacteriocin as inferred in previous studies (Schaller *et al*., 1981a,b; Harkness and Braun, 1990). Alternatively, the integrity of bacterial cell envelope is partially compromised by the bacteriocin due to the inhibition of biosynthesis of peptidoglycan polymer and the O‐antigen moiety of lipopolysaccharides (Schaller *et al*., 1982; Harkness and Braun, 1989a, b; Harkness and Braun, 1989b; Barreteau *et al*., 2010), which may lead to a detrimental increase in membrane permeability to Ca^2+^ and result in disruption of bacterial cell physiology. This hypothesis is based on the findings that bacterial cytosolic Ca^2+^ must be tightly controlled at low levels akin to those found in eukaryotic cells, and the disturbance of calcium ion homeostasis can affect bacterial survival (Dominguez, 2004; Miyamoto *et al*., 2005). In Gram‐negative bacteria, the periplasmic peptidoglycan polymer and the outer membrane lipopolysaccharides are crucial cell envelope components that are able to bind and prevent the entry of excessive divalent cations such as Mg^2+^ and Ca^2+^ into the cytoplasm (Jones *et al*., 2002; Silhavy *et al*., 2010; Clifton *et al*., 2014; Miller and Salama, 2018). Hence, an investigation on the change in levels of bacterial cytosol free Ca^2+^ in response to SyrM and Ca^2+^ treatments may help to provide further clues on the validity of this hypothesis.

We did not observe a significant reduction in bacterial population sizes when the *25D12* and *Pst* ^*ΔsaxE*^ mutant strains were subjected to growth assay in the presence of exudates or 1 mM calcium (Figs 1A–D, and 3A, C, D), suggesting that the SyrM is ineffective to kill the mutant strain under these culture conditions. However, we observed that higher levels of calcium supplemented to the medium (> 25 mM) substantially killed the *Pst* ^*ΔsaxE*^ mutant; likewise, when the *Pst* ^*ΔsaxE*^ mutant bacteria were infiltrated into plant leaves at higher levels (10^8^ CFU ml^−1^), a significant reduction of the bacterial population could be observed (not shown). Hence, the bactericidal activity of the SyrM protein is likely associated with high levels of ambient calcium and the density of bacterial producer, which are two features normally found in desiccated plant necrotic lesions. These observations further strengthen the pivotal role of SyrM in maintaining a predominant population during the necrotrophic stage of infection.

## Experimental procedures

### Bacterial strains and primers

Bacterial strains and primers used in this study are listed in Table S4 and S5 respectively.

### Construction of plasmids

Plasmids used in this study are listed in Table S6. For generation of pDEST6012, the reading frame cassette of pDEST17 (Invitrogen, Carlsbad, CA, USA) containing the *att*R sites, the chloramphenicol resistance gene (Cmr) and the *ccd*B gene was amplified by PCR and cloned into the SacI and XhoI sites of the plasmid pME6012 (Fan *et al*., 2011). To add the his‐tag to SaxE and SyrM, coding regions of *saxE* and *syrM* were amplified by PCR from genomic DNA of wild‐type *Pst* and recombined into pDONR201 (Invitrogen) to generate entry clones via Gateway Technology (Invitrogen). The coding sequence of 6xhis‐tag was added by PCR to the 3′ of the coding region of *saxE* and *syrM* genes on the pENTRY vectors respectively. The resulting pENTRY vectors were recombined with pDEST6012 and pDEST14 vector, respectively, to generate pME6012‐*saxEhis* and pDEST14‐*syrMhis*. PCR was used to insert an extra nucleotide into the *saxE orf* on pENTRY‐*saxEhis*, and the resulting vector was recombined with pDEST6012 to generate pME6012‐*saxEhis(ins)*. For generation of pME6012‐Cpl, the genomic region (630680–635203) of *Pst* used for complementation test was amplified by PCR and recombined into a pME6012DONR plasmid. The pME6012‐Cpl was further truncated with PstI, HindIII, SacI and XhoI, respectively, and the resulting products were self‐ligated to generate plasmids bearing the truncated regions for complementation test.

### Bacterial growth conditions

All *Pseudomonas syringae* strains were streaked on King's B (KB) plate (Fan *et al*., 2011) from −80°C stocks and grown overnight in KB medium at 28°C and washed once with sterilized water before use. Bacterial inoculum densities were as indicated in figure legends. For the assay of bacterial growth in the presence of leaf extracts or metal cation, 10 μl of bacterial suspension was mixed with 90 μl KB medium or KB containing the tested components in 96‐well plates and grown at 28°C, 200 rpm for 1 day. Cultures collected at indicated time points were serial‐diluted and spotted on KB plates supplemented with appropriate antibiotics for bacterial counts.

### Plant materials and bacterial inoculation

*Arabidopsis* and tomato plants were grown at 23°C with 9‐h light/15‐h dark cycle and 16‐h light/8‐h dark cycles respectively. Plant extracts were prepared as previously described (Fan *et al*., 2011). Five‐ to six‐week‐old *Arabidopsis* and tomato plants were used for bacterial infection experiments. For leaf infiltration assay, bacterial suspension mixed with 10 mM MgCl_2_ was infiltrated into the *Arabidopsis* and tomato plants at densities as indicated in figure legends. Unless otherwise noted, plants were covered with a plastic lid to keep high humidity for 1 day. For spray inoculation, plants were sprayed with bacterial suspension (OD_600_ = 0.2 in 10 mM MgCl_2_, 0.04% Silwet L‐77) and covered with a plastic lid to keep high humidity for 4 days.

### Bacterial growth assay under the desiccation–rehydration treatment

For the assay of bacterial growth on filter paper discs, 5 μl of bacterial suspensions was applied onto discs of 6 mm in diameter, and the inoculated discs were placed on KB plates overlaid with 0.5% soft agar (w/v) containing 1 mM CaCl_2_. After incubation at 28°C for 16 h, the discs were transferred to an empty petri dish (6 cm) and sealed in a bigger petri dish (15 cm) containing saturated solution of MgCl_2_ for desiccation treatment (32.5% relative humidity, RH) for 4 h at room temperature (Winston and Bates, 1960). After the treatment, paper discs were returned to a new soft agar plate to resume culture to the 48‐h time point at 28°C. Paper discs not subjected to desiccation treatment were used as controls.

For the assay of bacterial growth in leaf lesions, leaves of tomato plants were infiltrated with bacterial suspensions at indicated concentration and incubated at 25°C, 100% RH for 2 days. Discs of leaf lesion tissue were taken by a cork borer (6 mm in diameter) and transferred to an empty petri dish for desiccation treatment for 2 days as described above. For rehydration, the desiccated leaf lesion discs were misted with deionized water and incubated at 25°C, 100% RH for 2 days.

### Purification of the inhibitory component from *Arabidopsis* leaf extracts

Two hundred grams of *Arabidopsis* leaves was ground with liquid N_2_, mixed with 200 ml of water and incubated for 30 min before centrifugation at 10 000 rpm for 10 min. The resultant supernatant was boiled for 10 min and spin again to remove the pellet. After the addition of an equal amount of water into the supernatant, pass the solution through a column packed with 100 g of Dowex 1 resin. The resulting flow‐through was loaded on a weak cation exchanger column packed with 100 g of LEWATIT CNP‐105 resin and washed with 0.1 N NH_3_H_2_O. The column was eluted with 10% acetic acid, and the resulting fractions were collected for assays of inhibitory activity. Fractions with high inhibitory activity were freeze‐dried and dissolved with a minimal amount of deionized water. About 9 volume of methanol was added to the resulting solution to crystallize the active component.

### Purification of SyrM

An overnight culture of *E. coli* BL21 (DE3) carrying the plasmid pDEST14‐*syrMhis* was used to inoculate 2 l of LB broth at the ratio of 1:100. The inoculated broth was cultured at 37°C, 220 rpm till the OD_600_ reached 0.6 before the addition of 1 mM isopropyl *β*‐D‐thiogalactoside. The cells were grown for a further 12 h at 24°C and harvested by centrifugation at 4°C for 10 min at 6000 *g*. The cell pellet was resuspended in cold 20 mM Tris‐HCl buffer, pH 7.5, containing 500 mM NaCl and 5 mM imidazole, and disrupted by sonication using a Branson Digital Sonifier. The resulting suspension was centrifuged at 16 000 *g* for 30 min at 4°C. The cell‐free lysate was applied to nickel‐nitrilotriacetate (Ni^2+^‐NTA)‐agarose, and the protein was eluted over a 0–500 mM imidazole gradient. Purified protein was dialysed overnight into 20 mM Tris‐HCl, 100 mM NaCl, pH 8.0, was concentrated to 100 μM and then stored at −80°C after addition of 5% glycerol and filtration sterilization using a 0.22‐μm filter.

### Preparation of samples for SDS‐PAGE and protein gel blot analysis

For detection of his‐tagged SaxE in bacterial strains, cells were harvested by centrifugation at 10 000 *g* for 10 min at 4°C. The supernatant was removed and bacterial pellet was resuspended in protein loading buffer (50 mM Tris‐HCl, pH 7.5, 2% SDS, 0.1% bromphenol blue, 10% glycerol). Samples were then boiled for 10 min at 100°C and centrifuged at 10 000 *g* for 10 min at 4°C. The resulting supernatant was used for SDS‐PAGE. For detection of his‐tagged SyrM from inoculated filter paper discs, 100 μl of sterilized water was used to vortex and wash off bacteria on paper discs in a 1.5‐ml test tube. The resultant bacterial solutions were subjected to centrifugation, and supernatants were used for protein electrophoresis. For protein gel blot assay, proteins were transferred from the gels onto nitrocellulose membranes at 200 mA for 1.5 h. The membrane was blocked by TBST containing 5% milk powder and probed with mouse anti‐His antibody (1:2000; Sigma, Saint Louis, MO, USA). Goat anti‐Mouse‐HRP (1:10 000; Sigma) was used as secondary antibody. Blots were developed with the enhanced chemiluminescence (ECL) detection system (GE Healthcare, Milwaukee, WI, USA).

### Cytotoxicity assay of SyrM

Soft agar overlay method was used for determining the bactericidal activity of SyrM as described (Grinter *et al*., 2012) with minor modification. Five microlitres of 10 μM purified syringacin M protein was applied on a KB plate overlaid with 3 ml of molten soft agar inoculated with 100 μl of bacteria (OD_600_ = 0.5) as indicated. Bactericidal activity of SyrM was determined based on the sizes of inhibition zones. Results of all experiments were observed or measured at 1 dpi.

### Determination of free Ca^2+^ levels in the intercellular fluids of tomato leaves

Leaves infiltrated with wild‐type *Pst* were harvested by cutting petioles with a razor blade, carefully transferred into a 50‐ml centrifuge tube with xylem wound facing upward and centrifuged at 2000 *g* for 2 min at 4°C. The fluids were collected for measurement of free Ca^2+^ with a Calcium Colorimetric Assay Kit (NJJC Bioengineering Institute, Nanjing, China) following manufacturer's instructions. Ten microlitres of intercellular fluid was added into 250 μl solutions containing methylthymol blue, and OD_610_ was measured by Spectra Max i3x (Molecular Devices, Sunnyvale, CA, USA) after incubation for 5 min. Levels of free Ca^2+^ in intercellular fluids were calculated by an established standard curve.

### Quantitative real‐time PCR (qRT‐PCR) assay of *syrM* gene expression

Total RNA was isolated from bacteria on paper discs with TRIzol reagent (Invitrogen) following the manufacturer's instructions. Recombinant RNase‐free DNase I (Takara, Kusatsu, Japan) and SuperScript III Reverse Transcriptase (Invitrogen) were used to remove genomic DNA and synthesize the first‐strand cDNA respectively. Tenfold diluted solutions of the reaction products were used for the qRT‐PCR assay. The qRT‐PCR was performed in 12 μl volume with Bestar^®^ SYBR Green qPCR MasterMix (DBI^®^ Bioscience, Ludwigshafen, Germany) on ABI QuantStudio 6 Flex (Thermo Fisher, Waltham, MA, USA).

### Transposon mutagenesis and thermal asymmetric interlaced PCR (TAIL‐PCR) assay

*Pst* ^*▵saxAB/F*^ was mutagenized by electroporation with the suicide plasmid pEJL428 carrying the transposon Omegon‐Km (Ω‐Km; Joseph‐Liauzun *et al*., 1989; Fan *et al*., 2011), and the position of Ω‐Km was determined by TAIL‐PCR (Liu and Whittier, 1995).

## Conflict of interest

None declared.

## Supporting information


**Fig. S1.** *Pst* mutant *25D12* grew normally in KB medium.
**Fig. S2.** Ca^2+^ is the inhibitory factor that suppressed the growth of *Pst* strain deficient in the *saxE* gene.
**Fig. S3.** Ca^2+^ enhanced the relative transcript levels of *PSPTO_0572* gene.
**Fig. S4.** Bacterial populations in leaf lesions fluctuated widely during a desiccation‐rehydration regimen.
**Table S1.** Similarity of SaxE‐like hypothetical proteins in *Pseudomonas* strains.
**Table S2.** Sensitivity of the tested *P. syringae* strains to syringacin M.
**Table S3.** SyrM‐like sequences in *Pseudomonas* and other *Proteobacteria* strains.
**Table S4.** Bacterial strains used in this study.
**Table S5.** Primers used in this study.
**Table S6.** Plasmids used in this study.
