# Supplementary material for: Pseudomonas bacteriocin syringacin M released upon desiccation suppresses the growth of sensitive bacteria in plant necrotic lesions
Source: Microb Biotechnol. 2019 Jan 22;13(1):134–47. doi: 10.1111/1751-7915.13367 (PMC6922522; doi:10.1111/1751-7915.13367)
Supplement: Supplementary file 1 — Fig. S1. Pst mutant 25D12 grew normally in KB medium. Fig. S2. Ca2+ is the inhibitory factor that suppressed the growth of Pst strain deficient in the saxE gene. Fig. S3. Ca2+ enhanced the relative transcript levels of PSPTO_0572 gene. Fig. S4. Bacterial populations in leaf lesions fluctuated widely during a desiccation‐rehydration regimen. Table S1. Similarity of SaxE‐like hypothetical proteins in Pseudomonas strains. Table S2. Sensitivity of the tested P. syringae strains to syringacin M. Table S3. SyrM‐like sequences in Pseudomonas and other Proteobacteria strains. Table S4. Bacterial strains used in this study. Table S5. Primers used in this study. Table S6. Plasmids used in this study. [file MBT2-13-134-s001.docx]

**Table S1. Similarity of SaxE-like hypothetical proteins in *Pseudomonas* strains**

| **Strains** | **Accession number** | **Identity**^§^ |
| --- | --- | --- |
| *P. syringae* |  |  |
| pv. *antirrhini* ICMP4303 | KPW48562.1 | 100% |
| CC1417 | WP_024694425.1 | 44% |
| *P. fluorescens* |  |  |
| Pf29 | WP_030142638.1 | 30% |
| Q8r1-96 | EIK70830.1 | 26% |
| AG1429 | WP_114731548.1 | 26% |
| Pf275 | WP_109756029.1 | 24% |
| *P. brassicacearum* |  |  |
| NFM421 | AEA67278.1 | 26% |
| L13-6-12 | AOS39187.1 | 26% |
| DF41 | WP_049866950.1 | 26% |
| 51MFCVI2.1 | WP_028237202.1 | 25% |
| *P. brenneri* BS2771 | WP_090291371.1 | 22% |
| *Pseudomonas* sp. |  |  |
| NFACC37-1 | WP_092165361.1 | 26% |
| NFACC04-2 | SFW15059.1 | 26% |
| NFACC14 | SDX22901.1 | 26% |
| NFIX10 | SFA88917.1 | 25% |
| NFACC06-1 | SFE23969.1 | 26% |
| Pf153 | WP_053153867.1 | 25% |

SaxE-like sequences are searched by NCBI BLAST (<https://blast.ncbi.nlm.nih.gov/>). Identity^§^, amino acid sequences are compared with SaxE by DNA MAN8.

**Table S2. Sensitivity of the tested *P*. *syringae* strains to syringacin M**

| **Strains tested** | **Sensitivity** |
| --- | --- |
| *P. syringae* strains |  |
| pv. *maculicola* |  |
| M1 | - |
| M2 | - |
| M3 | - |
| M5 | - |
| M6 | - |
| ES4326 | - |
| pv. *syringae* |  |
| PSC1B | - |
| B64 | - |
| pv. *lachrymans* |  |
| 8 | ++ |
| NCPPB 540 | + |
| pv. *aptata* ptt G733 | - |
| pv. *api* 1089-5 | - |
| pv. *tomato* T1 | - |
| pv. *tabaci* 6605 | - |
| pv. *glycinea* race 4 | + |
| pv. *coronafaciens* KN221 | - |

Purified syringacin M protein were applied on a plate overlaid with soft agar inoculated with test strains shown above. Inhibition zones were observed at 1 dpi. -, no inhibitory effect. +, weak inhibitory effect. ++, strong inhibitory effect.

**Table S3. SyrM-like sequences in *Pseudomonas* and other *Proteobacteria* strains**

| **Strains** | **Accession number** | **Identity^§^** |
| --- | --- | --- |
| *Pseudomonas syringae* |  |  |
| pv. *tomato* | NP_790419.1 | 100% |
| pv. *persicae* | KKI23490.1 | 100% |
| pv. *maculicola* | KPB70694.1 | 100% |
| pv. *antirrhini* | KPW48536.1 | 100% |
| pv. *lachrymans* | EGH97664.1 | 99% |
| pv. *delphinii* | KPX22607.1 | 99% |
| pv. *theae* | EPM66381.1 | 99% |
| pv. *morsprunorum* | EGH12518.1 | 99% |
| pv. *atrofaciens* | KPW12251.1 | 95% |
| pv. *japonica* | EGH30336.1 | 94% |
| pv. *syringae* | ELS40993.2 | 94% |
| pv. *coryli* | WP_046237376.1 | 94% |
| pv. *lapsa* | ALU62567.1 | 94% |
| *Pseudomonas lundensis* | WP_094991005.1 | 54% |
| *Pseudomonas fluorescens* | WP_019694076.1 | 43% |
| *Pseudomonas aeruginosa* | WP_079990571.1 | 42% |
| *Pseudomonas brenneri* | WP_090291372.1 | 32% |
| *Pseudomonas brassicacearum* | WP_025213265.1 | 29% |
| *Salmonella enterica* | WP_079807659.1 | 42% |
| *Klebsiella pcneumoniae* | WP_047066220.1 | 42% |
| *Pantoea ananatis* | WP_050598374.1 | 40% |
| *Brenneria goodwinii* | WP_048639113.1 | 40% |
| *Phyllobacterium endophyticum* | WP_106715214.1 | 39% |
| *Pectobacterium carotovorum* | WP_014915624.1 | 38% |
| *Escherichia coli* | WP_106487363.1 | 38% |
| *Inquilinus limosus* | WP_052121549.1 | 38% |

SyrM-like sequences are searched by NCBI BLAST (<https://blast.ncbi.nlm.nih.gov/>). Identity^§^, amino acid sequences are compared with SyrM;

**Table S4. Bacterial strains used in this study**

| **Strains** | **Relative properties**^A^ | **Source/References** |
| --- | --- | --- |
| *P*. *syringae* strains |  |  |
| pv. *tomato* |  |  |
| DC3000 | WT *Pst*, Rif^R^ | Fan*, et al.*, 2011 |
| T1 | WT | Fan*, et al.*, 2011 |
| pv. *maculicola* |  |  |
| M1 | WT | Fan*, et al.*, 2011 |
| M2 | WT | Fan*, et al.*, 2011 |
| M3 | WT | Fan*, et al.*, 2011 |
| M5 | WT | Fan*, et al.*, 2011 |
| M6 | WT | Fan*, et al.*, 2011 |
| ES4326 | WT | Fan*, et al.*, 2011 |
| pv. *syringae* |  |  |
| PSC1B | WT | Fan*, et al.*, 2011 |
| B64 | WT | Fan*, et al.*, 2011 |
| pv. *lachrymans* |  |  |
| 8 | WT | From T.C. Zhao |
| NCPPB 540 | WT | From T.C. Zhao |
| pv. *apii* 1089-5 | WT | Fan*, et al.*, 2011 |
| pv. *aptata* ptt G733 | WT | Fan*, et al.*, 2011 |
| pv. *tabaci* 6605 | WT | Fan*, et al.*, 2011 |
| pv. *glycinea* race 4 | WT | Fan*, et al.*, 2011 |
| pv. *coronafaciens* KN221 | WT | Fan*, et al.*, 2011 |
| Constructed strains |  |  |
| *△saxE* | Deletion of *saxE orf* in *Pst* background | This study |
| *△syrM* | Deletion of *syrM* gene | This study |
| *△syrM△saxE* | Deletion of *syrM* and *saxE* | This study |
| *△saxAB/F* | Deletion of *saxA*, *saxB* and *saxF* | Fan*, et al.*, 2011 |
| *25D12* | Ω-Km insertion in*△saxAB/F* background, Rif^R^, Km^R^ | This study |
| *25D12*-Cpl | *25D12* carrying plasmid pME6012-Cpl, Rif^R^, Km^R^, Tet^R^ | This study |
| *25D12*-Cpl-PstI | *25D12* carrying pME6012-Cpl-PstI | This study |
| *25D12*-Cpl-HindIII | *25D12* carrying pME6012-Cpl-HindIII | This study |
| *25D12*-Cpl-SacI | *25D12* carrying pME6012-Cpl-SacI | This study |
| *25D12*-Cpl-XhoI | *25D12* carrying pME6012-Cpl-XhoI | This study |
| *Pst^lux^* | Insertion of *luxCDABE* in *Pst* background, Rif^R^, Km^R^ | Fan*, et al.*, 2008 |
| *△saxE^lux^* | Deletion of *saxE* in *Pst^lux^* background | This study |
| *△saxE-saxEhis* | *△saxE* carrying pME6012-*saxEhis*, Rif^R^, Tet^R^ | This study |
| *△saxE-saxEhis(ins)* | *△saxE* carrying pME6012-*saxEhis(ins)* | This study |
| *Psl*-pME6012 | *Psl* carrying pME6012, Tet^R^ | This study |
| *Psl*-pME6012-*saxEhis* | *Psl* carrying pME6012-*saxEhis* | This study |
| *E. coli* strain |  |  |
| *BL21(DE3)* |  | Thermo Fisher |
| *DEST14-syrMhis* | BL21 carrying pDEST14-*syrMhis*, Amp^R^ | This study |

^A^Abbreviations: R, resistance; Amp, ampicillin; Km, kanamycin; Rif, rifampicin; Tet, tetracycline.

**Table S5. Primers used in this study**

| **Primers** | **Sequence** | **Purpose** |
| --- | --- | --- |
| ΔsaxEjunctnFw | gattgacaatgtagcgtctgatttcaggcgctct | Deletion of *saxE* |
| ΔsaxEdwnstrm | agaaagctgggtcaagagcaaccgcagcccagtc |  |
| ΔsaxEjunctnRv | tgaaatcagacgctacattgtcaatccttatgga | Deletion of *saxE* |
| ΔsaxEupstrm | aaaaagcaggctccttcgcgaacatcaacttatt |  |
| ΔsyrMsaxEjunctnFw | acaggtaaaatcaatgtagcgtctgatttcag | Deletion of *syrM* and *saxE* |
| ΔsyrMsaxEdwnstrm | agaaagctgggtcaagagcaaccgcagcccagtc |  |
| ΔsyrMsaxEjunctnRv | aaaaagcaggcttattgttcggccttgagcggtgtg | Deletion of *syrM* and *saxE* |
| ΔsyrMsaxEupstrm | ctgaaatcagacgctacattgattttacctgt |  |
| ΔsyrMjunctnFw | ttattaacaggtaaaatcaatgtaggtcaaaggcatgaca | Deletion of *syrM* |
| ΔsyrMdwnstrm | agaaagctgggtgccgcctgggtggtggtttc |  |
| ΔsyrMjunctnRv | tgtcatgcctttgacctacattgattttacctgttaataa | Deletion of *syrM* |
| ΔsyrMupstrm | aaaaagcaggcttattgttcggccttgagcggtgtg |  |
| saxE for | aaaaagcaggctgttcggccttgagcggtgtg | Cloning of *saxE* |
| saxE rev | agaaagctgggtattgggcttcctcttggtta |  |
| syrM for | aaaaagcaggctagcggaatccttcgcgaaca | Cloning of *syrM* |
| syrM rev | agaaagctgggtcctgtcatgcctttgaccta |  |
| attB1 adapter | ggggacaagtttgtacaaaaaagcaggct | Generation of attB sites |
| attB2 adapter | ggggaccactttgtacaagaaagctgggt |  |
| KmTAIL-1 | aggctggctttttcttgttatcg | Flanking sequence of Ω-Km |
| KmTAIL-2 | gtggatgaccttttgaatgacc | Flanking sequence of Ω-Km |
| qsyrM for | tcagagcgcacgcccagttg | qRT-PCR of *syrM* |
| qsyrM rev | agtgtaatgttccccagcgtcagc |  |
| qrecA for | aggcaagtatttcctgtcgcc | qRT-PCR of *recA* |
| qrecA rev | ctggtactcacccagcagttttt |  |

**Table S6. Plasmids used in this study**

| **Plasmids** | **Relative propertiesA** | **Source/References** |
| --- | --- | --- |
| pME6012 | Constitutive expression vector ,Tet^R^ | Heeb*, et al.*, 2000 |
| pDEST6012 | Destination vector modified from pME6012 | This study |
| pME6012-*saxEhis* | Constitutive expression of *saxE-6×his* | This study |
| pME6012-*saxEhis(ins)* | *saxE orf* deficiency in pME6012-*saxEhis* | This study |
| pME6012-*syrMhis* | Constitutive expression of *syrM-6×his* | This study |
| pME6012-Cpl | Genomic region complementary vector^₤^ | This study |
| pME6012-Cpl-PstI | Genomic region complementary vector^₤^ | This study |
| pME6012-Cpl-HindIII | Genomic region complementary vector^₤^ | This study |
| pME6012-Cpl-SacI | Genomic region complementary vector^₤^ | This study |
| pME6012-Cpl-XhoI | Genomic region complementary vector^₤^ | This study |
| pDEST14 | T7 promotor destination vector, Amp^R^ | Invitrogen |
| pDEST14-*syrMhis* | Inducible expression of *syrM-6×his* | This study |

^A^Abbreviations: R, resistance; Amp, ampicillin; Tet, tetracycline.

₤, complementary region as indicated in Fig. 2.


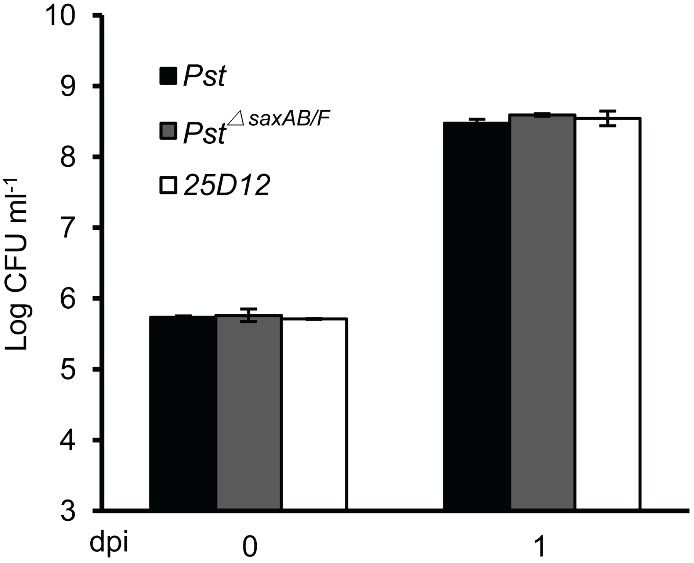


**Fig. S1. *Pst* mutant *25D12* grew normally in KB medium.**

Wild type *Pst*, *Pst^△saxAB/F^* and *25D12* strains were inoculated into the KB medium at OD_600_=0.001. Samples were collected at 0 and 1 dpi for colony counts. All experiments were repeated at least two times and similar results were observed. Data shown are means ± standard deviation.


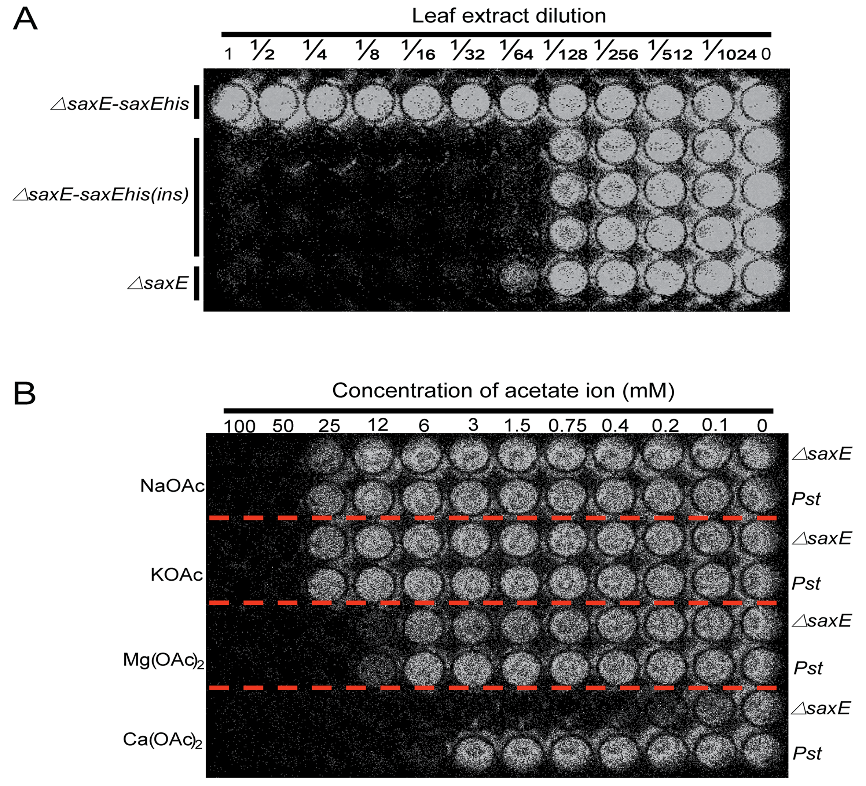


**Fig. S2. Ca^2+^ is the inhibitory factor that suppressed the growth of *Pst* strain deficient in the *saxE* gene.**

(A) Plasmid pME6012 expressing his-tagged wild type SaxE or mutated SaxEins were introduced into the *Lux*-tagged *△saxE* strain. Resulting strains, as well as the *△saxE* strain carrying empty vector control, were inoculated at OD600=0.001 into serial diluted leaf extracts in a 96-well plate. The inoculated plate was shaken at 200 rpm at 28℃ for 1 d. Bacterial growth was visualized by the luminescence of the cultures in each well. (B) *Lux*-tagged wild type *Pst*, or *△saxE* were inoculated into the KB medium containing serial diluted solutions of acetate salts at OD_600_=0.001. Bacterial growth was visualized as in (A).


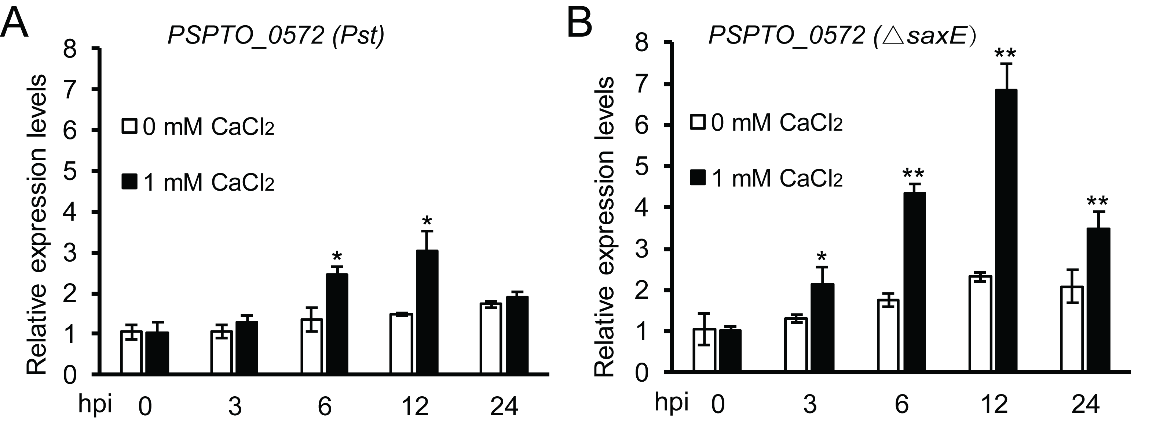


**Fig. S3. Ca^2+^ enhanced the relative transcript levels of *PSPTO_0572* gene.**

Wild type *Pst* and *△saxE* mutant strain were inoculated into liquid KB media with or without 1 mM CaCl_2_ at OD_600_=0.1. Total RNAs were prepared from samples collected at indicated time points and subjected to the quantitative RT-PCR assay of the transcript levels of *PSPTO_0572*. The *recA* was used as the reference gene. All experiments were repeated at least three times and similar results were observed. Data shown are means ± standard deviation. **and * indicate significant difference (*t*-test), p<0.01 and p<0.05, respectively.


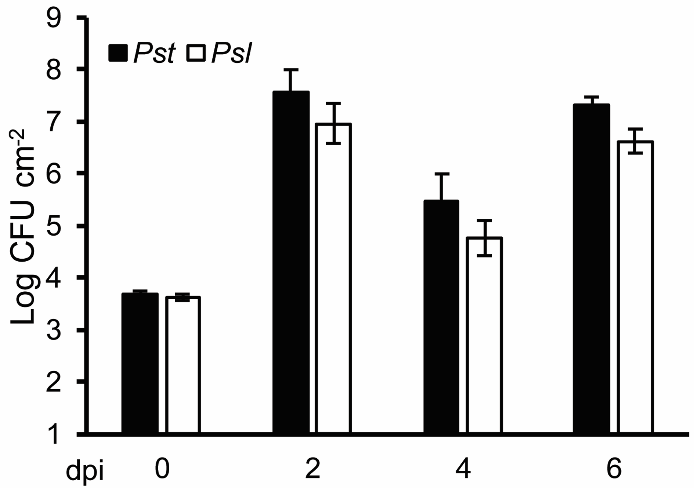


**Fig. S4. Bacterial populations in leaf lesions fluctuated widely during a desiccation-rehydration regimen.**

*Pst* and *Psl* were separately infiltrated into tomato leaves at OD_600_=0.001. Samples were treated with the same conditions as in Fig. 6C. Levels of bacterial population sizes were investigated at indicated time points. All experiments were repeated at least three times and similar results were observed. Data shown are means ± standard deviation.

**References**

Fan, J., Crooks, C., Creissen, G., Hill, L., Fairhurst, S., Doerner, P., and Lamb, C. (2011) *Pseudomonas sax* genes overcome aliphatic isothiocyanate–mediated non-host resistance in Arabidopsis. *Science* **331**: 1185-1188.

Fan, J., Crooks, C., and Lamb, C. (2008) High-throughput quantitative luminescence assay of the growth *in planta* of *Pseudomonas syringae* chromosomally tagged with *Photorhabdus luminescens luxCDABE*. *Plant J* **53**: 393-399.

Heeb, S.Y., Nishijyo, T., Schnider, U., Keel, C., Wade, J., Walsh, U.*, et al.* (2000) Small, stable shuttle vectors based on the minimal pVS1 replicon for use in gram-negative, plant-associated bacteria. *Mol Plant Microbe Interact* **13**: 232-237.
